# Meta-Strategy for Learning Tuning Parameters with Guarantees

**DOI:** 10.3390/e23101257

**Published:** 2021-09-27

**Authors:** Dimitri Meunier, Pierre Alquier

**Affiliations:** 1 Istituto Italiano di Tecnologia, 16163 Genoa, Italy; dimitri.meunier.21@ucl.ac.uk; 2 RIKEN Center for Advanced Intelligence Project, Tokyo 103-0027, Japan

**Keywords:** meta-learning, hyperparameters, priors, online learning, Bayesian inference, online optimization, gradient descent

## Abstract

Online learning methods, similar to the online gradient algorithm (OGA) and exponentially weighted aggregation (EWA), often depend on tuning parameters that are difficult to set in practice. We consider an online meta-learning scenario, and we propose a meta-strategy to learn these parameters from past tasks. Our strategy is based on the minimization of a regret bound. It allows us to learn the initialization and the step size in OGA with guarantees. It also allows us to learn the prior or the learning rate in EWA. We provide a regret analysis of the strategy. It allows to identify settings where meta-learning indeed improves on learning each task in isolation.

## 1. Introduction

In many applications of modern supervised learning, such as medical imaging or robotics, a large number of tasks is available but many of them are associated with a small amount of data. With few datapoints per task, learning them in isolation would give poor results. In this paper, we consider the problem of learning from a (large) sequence of regression or classification tasks with small sample size. By exploiting their similarities we seek to design algorithms that can utilize previous experience to rapidly learn new skills or adapt to new environments.

Inspired by human ingenuity in solving new problems by leveraging prior experience, *meta-learning* is a subfield of machine learning whose goal is to automatically adapt a learning mechanism from past experiences to rapidly learn new tasks with little available data. Since it “learns the learning mechanism” it is also referred to as *learning-to-learn* [1]. It is seen as a critical problem for the future of machine learning [2]. Numerous formulations exist for meta-learning and we focus on the problem of *online meta-learning* where the tasks arrive one at a time and the goal is to efficiently transfer information from the previous tasks to the new ones such that we learn the new tasks as efficiently as possible (this has also been refered to as *lifelong learning*). Each task is in turn processed *online*. To sum up, we have a stream of tasks and for each task a stream of observations.

In order to solve online tasks, diverse well-established strategies exist: perceptron, online gradient algorithm (OGA), online mirror descent, follow-the-regularized-leader, exponentially weighted aggregation (EWA, also refered to as *generalized Bayes* etc.) We refer the reader to [3,4,5,6] for introductions to these algorithms and to so-called regret bounds, that control their generalization errors. We refer to these algorithms as the *within-task* strategies. The big challenge is to design a meta-strategy that uses past experiences to adapt a within-task strategy to perform better on the next tasks.

In this paper, we propose a new meta-learning strategy. The main idea to learn the tuning parameters is to minimize its regret bound. We provide a meta-regret analysis for our strategy. We illustrate our results in the case where the within-task strategy is the online gradient algorithm, and exponentially weighted aggregation. In the case of OGA, the tuning parameters considered are the initialization and the gradient steps. For EWA, we consider either the learning rate, or the prior. In each case, we compare the regret incurred when learning the tasks in isolation to our meta-regret bound. This allows us to identify settings where meta-learning indeed improves on learning in isolation.

### 1.1. Related Works

Meta-learning is similar to multitask learning [7,8,9] in the sense that the learner faces many tasks to solve. However, in multitask learning, the learner is given a fixed number of tasks, and can learn the connections between these tasks. In meta-learning, the learner must prepare to face future tasks that are not given yet.

Meta-learning is often referred to as learning-to-learn or lifelong learning. The authors of [10] proposed the following distinction: “learning-to-learn” for situations where the tasks are presented simultaneously, and “lifelong learning” for situations where they are presented sequentially. Following this terminology, learning-to-learn algorithms were proposed very early in the literature, with generalization guarantees [11,12,13,14,15,16].

On the other hand, in the lifelong learning scenario, until recently, algorithms were proposed without generalization guarantees [17,18]. A theoretical study was proposed by [10], but the strategies in that paper are not feasible in practice. This problem was recently improved [19,20,21,22,23,24,25,26]. In a similar context, in [27], the authors propose an efficient strategy to learn the starting point of OGA. However, an application of this strategy to learning the step size do not show any improvement over learning in isolation [28]. The closest work to this paper is [29] in which they also suggest a regret bound minimization strategy. This paper indeed provides a meta-regret bound for learning both the initialization and the gradient step. Note, however, that this paper remains specific to OGA, while our work can be potentially applied to any online learning algorithm. Indeed, we provide another example: the generalized Bayesian algorithm EWA, for which we learn the prior, or the learning rate. To learn the prior is new in the online setting, to our knowledge. It can be related to works in the batch setting [11,13,15,16], but the improvement with respect to learning in isolation is not quantified in these works.

Finally, it is important to note that we focus on the case where the number of tasks *T* is large, while the sample size *n* and algorithmic complexity of each task is moderately small. When each task is extremely complex, for example training a deep neural network on a huge dataset, our procedure (as well as those discussed above) will become too expansive. Alternative approaches were proposed, based on optimization via multi-armed bandits [30,31].

### 1.2. Organization of the Paper

In Section 2, we introduce the formalism of meta-learning and the notations that will be used throughout the paper. In Section 3, we introduce our meta-learning strategy, and its theoretical analysis. In Section 4, we provide the details of our method in the case of meta-learning the initialization and the step size in the online gradient algorithm. Based on our theoretical results, there are also explicit situations where meta-learning indeed improves on learning the tasks independently. This is confirmed by experiments reported in this section. In Section 5, we provide the details of our methodology when the algorithm used within tasks is a generalized Bayesian algorithm: EWA. We show how our meta-strategy can be used to tune the learning rate; we also discuss how it can be used to learn priors. The proofs of the main results are given in Section 6.

## 2. Notations and Preliminaries

By convention, vectors $v\in R^{d}$ are seen as d×1 matrices (columns). Let ∥v∥ denote the Euclidean norm of *v*. Let $A^{T}$ denote the transposition of any d×k matrix *A*, and $I_{d}$ the d×d identity matrix. For two real numbers *a* and *b*, let a∨b=max(a,b) and a∧b=min(a,b). For z∈R, $z_{+}$ is its positive part $z_{+}=z\vee 0$. Given a finite set *S*, we let card(S) denote the cardinality of *S*.

The learner has to solve tasks t=1,…,T sequentially. Each task *t* consists in *n* rounds i=1,…,n. At each round *i* of task *t*, the learner has to take a decision $\theta_{t,i}$ in a decision space $\Theta \subseteq R^{d}$ for some d>0. Then, a convex loss function $\ell_{t,i}:\Theta \to R$ is revealed to the learner, who incurs the loss $\ell_{t,i}\left(\theta_{t,i}\right)$. Classical examples with $\Theta \subset R^{d}$ include regression tasks, where $\ell_{t,i}\left(\theta \right)={\left(y_{t,i}-x_{t,i}^{T}\theta \right)}^{2}$ for some $x_{t,i}\in R^{d}$ and $y_{t,i}\in R$. For classification tasks, $\ell_{t,i}\left(\theta \right)={\left(1-y_{t,i}x_{t,i}^{T}\theta \right)}_{+}$ for some $x_{t,i}\in R^{d}$, $y_{t,i}\in \left\{-1,+1\right\}$.

**Figure 1:**
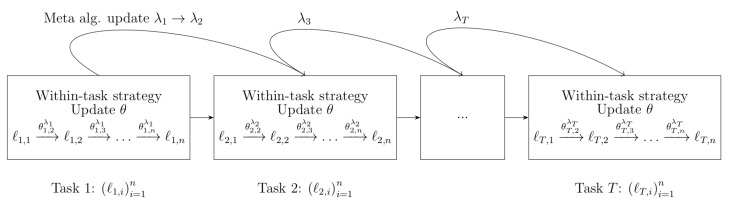
The dynamics of meta-learning.

Throughout the paper, we will assume that the learner uses, for each task, an online decision strategy called *within-task strategy*, parametrized by a tuning parameter λ∈Λ where Λ is a closed, convex subset of $R^{p}$ for some p>0. Example of such strategies include the online gradient algorithm, given by $\theta_{t,i}=\theta_{t,i-1}-\gamma \nabla \ell_{t,i}\left(\theta_{t,i-1}\right)$. In this case, the tuning parameters are the initialization, or starting point, $\theta_{t,1}=\vartheta$ and the learning rate, or step size, γ. That is, λ=(ϑ,γ), so p=d+1. The parameter λ is kept fixed during the whole task. It is of course possible to use the same parameter λ in *all* the tasks. However, we will be interested here in defining *meta-strategies* that will allow us to improve λ task after task, based on the information available so far. In Section 3, we will define such strategies. For now, let $\lambda_{t}$ denote the tuning parameter used by the learner all along task *t*. Figure 1 provides a recap of all the notations.

Let $\theta_{t,i}^{\lambda }$ denote the decision at round *i* of task *t* when the online strategy is used with parameter λ. We will assume that a regret bound is available for the within-task strategy. By this, we mean that there is a set $\Theta_{0}\subset \Theta$ of parameters of interest, and that the learner knows a function $B_{n}:\Theta \times \Lambda \to R$ such that, for any task *t*, for any λ∈Λ,

(1) $$\sum_{i=1}^{n}\ell_{t,i}\left(\theta_{t,i}^{\lambda }\right)\leq \underset{=:L_{t}\left(\lambda \right)}{\underset{︸}{\underset{\theta \in \Theta_{0}}{\inf}\left\{\sum_{i=1}^{n}\ell_{t,i}\left(\theta \right)+B_{n}\left(\theta ,\lambda \right)\right\}}}.$$

For OGA, regret bounds can be found, for example, in [4,6] (in this case, $\Theta_{0}=\Theta$). Other examples include exponentially weighted aggregation (bounds in [3], here $\Theta_{0}$ is a finite set of predictors while decisions Θ are probability distributions on $\Theta_{0}$). More examples will be discussed in the paper. For a fixed parameter θ, the quantity $\sum_{i=1}^{n}\ell_{t,i}\left(\theta_{t,i}^{\lambda }\right)-\sum_{i=1}^{n}\ell_{t,i}\left(\theta \right)$ measures the difference between the total loss suffered during task *t*, and the loss what one would have suffered using the parameter θ. It is thus called “the regret with respect to parameter θ”, and $B_{n}\left(\theta ,\lambda \right)$ is usually referred to as a “regret bound”. We will call $L_{t}\left(\lambda \right)$ the “meta-loss”. In [29], the authors study a meta-strategy that minimizes the meta-loss of OGA. Indeed, if (1) is tight, to minimize the right-hand side is a good way to ensure that the left-hand side, that is, the cumulated loss, is small. In this work, we will focus on meta-strategies minimizing the meta-loss in a more general context.

The simplest meta-strategy is learning in isolation. That is, we keep $\lambda_{t}=\lambda_{0}\in \Lambda$ for all tasks. The total loss after task *T* is then given by:

(2) $$\sum_{t=1}^{T}\sum_{i=1}^{n}\ell_{t,i}\left(\theta_{t,i}^{\lambda_{0}}\right)\leq \sum_{t=1}^{T}L_{t}\left(\lambda_{0}\right).$$

However, when the learner uses a meta-strategy to improve the tuning parameter at the end of each task, the total loss is given by $\sum_{t=1}^{T}\sum_{i=1}^{n}\ell_{t,i}\left(\theta_{t,i}^{\lambda_{t}}\right)$. We will, in this paper, investigate strategies with meta-regret bounds; that is, bounds of the form

(3) $$\sum_{t=1}^{T}\sum_{i=1}^{n}\ell_{t,i}\left(\theta_{t,i}^{\lambda_{t}}\right)\leq \underset{\lambda \in \Lambda }{\inf}\left\{\sum_{t=1}^{T}L_{t}\left(\lambda \right)+C_{T}\left(\lambda \right)\right\}.$$

Of course, such bounds will be relevant only if the right-hand side of (3) is not larger than the right-hand side of (2), and is significantly smaller in some favourable settings. We show when this is the case in Section 4.

## 3. Meta-Learning Algorithms

In this section, we provide two meta-strategies to update λ at the end of each task. The first one is a direct application of OGA to meta-learning. It is computationally simpler, but feasible only in the special case where we have an explicit formula for the (sub-)gradient of each $L_{t}\left(\lambda \right)$. The second one is an application of implicit online learning to our setting. In Section 4, we provide an example where this is the case. The second meta-strategy can be used without this assumption. In both cases, we provide a regret bound as (3), under the following condition.

**Assumption** **1.** *For any t∈{1,…,T}, the function $\lambda \mapsto L_{t}\left(\lambda \right)$ is L-Lipschitz and convex.*

### 3.1. Special Case: The Gradient of the Meta-Loss Is Available in Closed Form

As each $L_{t}$ is convex, its subdifferential at each point of Λ is non-empty. For the sake of simplicity, we will use the notation $\lambda \mapsto \nabla L_{t}\left(\lambda \right)$ in the following formulas to denote *any* element of its subdifferential at λ. We define the online gradient meta-strategy (OGMS) with step α>0 and starting point $\lambda_{1}\in \Lambda$: for any t>1,

(4) $$\lambda_{t}=\Pi_{\Lambda }\left[\lambda_{t-1}-\alpha \nabla L_{t-1}\left(\lambda_{t-1}\right)\right]$$

where $\Pi_{\Lambda }$ denotes the orthogonal projection on Λ.

### 3.2. The General Case

We now cover the general case, where a formula for the gradient of $L_{t}\left(\lambda \right)$ might not be available. We propose to apply a strategy that was first defined in [32] for online learning, and studied under the name “implicit online learning” (we refer the reader to [33] and the references therein). In the meta-learning context, this gives the online proximal meta-strategy (OPMS) with step α>0 and starting point $\lambda_{1}\in \Lambda$, defined by:

(5) $$\lambda_{t}=\underset{\lambda \in \Lambda }{\mathrm{argmin}}\left\{L_{t-1}\left(\lambda \right)+\frac{∥\lambda -\lambda_{t-1}{∥}^{2}}{2\alpha }\right\}.$$

Using classical notations, e.g., [34], we can rewrite this definition with the proximal operator (hence the name of the method). Indeed $\lambda_{t}={\mathrm{prox}}_{\alpha L_{t-1}}\left(\lambda_{t-1}\right)$ where prox is the proximal operator given for any x∈Λ and any convex function f:Λ→R,

(6) $${\mathrm{prox}}_{f}\left(x\right)=\underset{\lambda \in \Lambda }{\mathrm{argmin}}\left\{f\left(\lambda \right)+\frac{{∥x-\lambda ∥}^{2}}{2}\right\}.$$

This strategy is feasible in practice in the regime we are interested in; that is, when *n* is small or moderately large, and T→∞. The learner has to store all the losses of the current task $\ell_{t-1,1},\ldots ,\ell_{t-1,n}$. At the end of the task, the learner can use any convex optimization algorithm to minimize, with respect to (θ,λ)∈Θ×Λ, the function

(7) $$F_{t}\left(\theta ,\lambda \right)=\sum_{i=1}^{n}\ell_{t,i}\left(\theta \right)+B_{n}\left(\theta ,\lambda \right)+\frac{∥\lambda -\lambda_{t-1}{∥}^{2}}{2\alpha }.$$

We can use a (projected) gradient descent on $F_{t}$ or its accelerated variants [35].

### 3.3. Regret Analysis

A direct application of known results to the setting of this paper leads to the following proposition. For the sake of completeness, we still provide the proofs in Section 6.

**Proposition** **1.** *Under Assumption 1, using either OGMS or OPMS with step α>0 and starting point $\lambda_{1}\in \Lambda$ leads to*

(8) $$\sum_{t=1}^{T}\sum_{i=1}^{n}\ell_{t,i}\left(\theta_{t,i}^{\lambda_{t}}\right)\leq \underset{\lambda \in \Lambda }{\inf}\left\{\sum_{t=1}^{T}L_{t}\left(\lambda \right)+\frac{\alpha TL^{2}}{2}+\frac{∥\lambda -\lambda_{1}{∥}^{2}}{2\alpha }\right\}.$$

The proof can be found in Section 6.

## 4. Example: Learning the Tuning Parameters of Online Gradient Descent

In all this section, we work under the following condition.

**Assumption** **2.** *For any (t,i)∈{1,…,T}×{1,…,n}, the function $\ell_{t,i}$ is* Γ-*Lipschitz and convex*.

### 4.1. Explicit Meta-Regret Bound

We study the situation where the learner uses (projected) OGA as a within-task strategy; that is, $\Theta =\{\theta \in R^{d}:∥\theta ∥\leq C\}$ and, for any i>1,

(9) $$\theta_{t,i}=\Pi_{\Theta }\left[\theta_{t,i-1}-\gamma \nabla \ell_{t,i}\left(\theta_{t,i-1}\right)\right].$$

With such a strategy, we already mentioned that $\lambda =\left(\vartheta ,\gamma \right)\in \Lambda \subset \Theta \times R_{+}$ contains an initialization and a step size. An application of the results in Chapter 11 in [3] gives $B_{n}\left(\theta ,\lambda \right)=B_{n}\left(\theta ,\left(\vartheta ,\gamma \right)\right)=\gamma \Gamma^{2}n/2+{∥\theta -\vartheta ∥}^{2}/\left(2\gamma \right)$. So

(10) $$L_{t}\left(\left(\vartheta ,\gamma \right)\right)=\underset{∥\theta ∥\leq C}{\inf}\left\{\sum_{i=1}^{n}\ell_{t,i}\left(\theta \right)+\frac{\gamma \Gamma^{2}n}{2}+\frac{{∥\theta -\vartheta ∥}^{2}}{2\gamma }\right\}.$$

It is quite direct to check Assumption 1. We summarize this in the following proposition.

**Proposition** **2.** *Under Assumption 2, assume that the learner uses OGA as an inner algorithm. Assume $\Lambda =\{\vartheta \in R^{d}:∥\vartheta ∥\leq C\}\times \left[\underline{\gamma },\bar{\gamma }\right]$ for some C>0 and $0<\underline{\gamma }<\bar{\gamma }<\infty$. Then Assumption 1 is satisfied with*

(11) $$L:=\sqrt{\frac{n^{2}\Gamma^{4}}{4}+\frac{4C^{2}}{{\underline{\gamma }}^{2}}+\frac{4C^{4}}{{\underline{\gamma }}^{4}}}.$$

So, when the learner uses one of the meta-strategies OGMS or OPMS, we can apply Proposition 1 respectively. This leads to the following theorem.

**Theorem** **1.** *Under the assumptions of Proposition 2, with $\underline{\gamma }=1/n^{\beta }$ for some β>0 and $\bar{\gamma }=C^{2}$, when the learner uses either OGMS or OPMS with*

(12) $$\alpha =\frac{C}{L}\sqrt{\frac{4+C^{2}}{T}}$$

*(where L is given by (11)), we have:*

(13) $$\sum_{t=1}^{T}\sum_{i=1}^{n}\ell_{t,i}\left(\theta_{t,i}^{\lambda_{t}}\right)\leq \underset{\theta_{1},\ldots ,\theta_{T}\in \Theta }{\inf}\left\{\sum_{t=1}^{T}\sum_{i=1}^{n}\ell_{t,i}\left(\theta_{t}\right)+C(\Gamma ,C)\left[n^{1\vee 2\beta }\sqrt{T}+\left(n^{1-\beta }+\sigma \left(\theta_{1}^{T}\right)\sqrt{n}\right)T\right]\right\}$$

*where C(Γ,C)>0 depends only on (Γ,C) and where:*

(14) $$\sigma \left(\theta_{1}^{T}\right)=\sqrt{\frac{1}{T}\sum_{t=1}^{T}{∥\theta_{t}-\frac{1}{T}\sum_{s=1}^{T}\theta_{s}∥}^{2}}.$$

Let us compare this result with learning in isolation, as defined in (2); that is, solving the sequence of tasks with a constant hyperparameter λ=(ϑ,γ). For the usual choice ϑ=0 and $\gamma =c/\sqrt{n}$ where *c* is a constant that does not depend on *n* nor *T*, OGA leads to a regret in $O(\sqrt{n})$. After *T* tasks, learning in isolation thus leads to a regret in $T\sqrt{n}$. Our strategies with β=1 lead to a regret in

(15) $$n^{2}\sqrt{T}+\left(1+\sigma \left(\theta_{1}^{T}\right)\sqrt{n}\right)T.$$

The term $n^{2}\sqrt{T}$ is the price to pay for meta-learning. In the regime we are interested in (small *n*, large *T*), which is smaller than $T\sqrt{n}$. Consider the leading term. In the worst case scenario, this is also $T\sqrt{n}$. However, there are good predictors $\theta_{1},\ldots ,\theta_{T}$ for tasks 1,…,T, respectively, such that $\sigma (\theta_{1}^{T})$ is small, and in this case we see the improvement with respect to learning in isolation. The extreme case is when there is a good predictor $\theta^{*}$ that predicts well for all tasks. In this case, regret with respect to $\theta_{1}=\ldots =\theta_{T}=\theta^{*}$ is in $n^{2}\sqrt{T}+T$, which improves significantly on learning in isolation. Note however that, using a different meta-strategy, specifically designed for OGA, Ref. [29] obtain a better dependence on *T* when $\sigma (\theta_{1}^{T})=0$.

Let us now discuss the implementation of our meta-stategy. We first remark that under the quadratic loss, it is possible to derive a formula for $L_{t}$, which allows to use OGMS. We then discuss OPMS for the general case.

### 4.2. Special Case: Quadratic Loss

First, consider $\ell_{t,i}={\left(y_{t,i}-x_{t,i}^{T}\theta \right)}^{2}$ for some $y_{t,i}\in R$ and $x_{t,i}\in R^{d}$. Assumption 2 is satisfied if we assume, moreover that all $|y_{t,i}|\leq c$ and $∥x_{t,i}∥\leq b$, with $\Gamma =2bc+2b^{2}C$. In this case,

(16) $$L_{t}\left(\left(\vartheta ,\gamma \right)\right)=\underset{∥\theta ∥\leq C}{\inf}\left\{\sum_{i=1}^{n}{\left(y_{t,i}-x_{t,i}^{T}\theta \right)}^{2}+\frac{\gamma \Gamma^{2}n}{2}+\frac{{∥\theta -\vartheta ∥}^{2}}{2\gamma }\right\}.$$

Define $Y_{t}={\left(y_{t,1},\ldots ,y_{t,n}\right)}^{T}$ and $X_{t}=(x_{t,1}\left|\ldots \right|x_{t,n}{)}^{T}$. The minimizer of $\sum_{i=1}^{n}{\left(y_{t,i}-x_{t,i}^{T}\theta \right)}^{2}+{∥\theta -\vartheta ∥}^{2}/\left(2\gamma \right)$ with respect to θ is known as the ridge regression estimator:

(17) $${\hat{\theta }}_{t}={\left(X_{t}^{T}X_{t}+\frac{I_{d}}{2\gamma }\right)}^{-1}\left(X_{t}^{T}Y_{t}+\frac{\vartheta }{2\gamma }\right).$$

This also coincides with the minimizer in the right-hand side of (16) on the condition that $∥{\hat{\theta }}_{t}∥\leq C$. In this case, by plugging ${\hat{\theta }}_{t}$ in (16), we have a close form formula for $L_{t}\left(\left(\vartheta ,\gamma \right)\right)$, and an explicit (but cumbersome) formula for its gradient. It is thus possible to use the OGMS strategy to update λ=(ϑ,γ).

### 4.3. The General Case

In the general case, denote $\lambda_{t-1}=\left(\vartheta_{t-1},\gamma_{t-1}\right)$, then $\lambda_{t}=\left(\vartheta_{t},\gamma_{t}\right)$ is obtained by minimizing

(18) $$F_{t}\left(\theta ,\left(\vartheta ,\gamma \right)\right)=\sum_{i=1}^{n}\ell_{t,i}\left(\theta \right)+\frac{\gamma \Gamma^{2}n}{2}+\frac{{∥\theta -\vartheta ∥}^{2}}{2\gamma }+\frac{∥\vartheta -\vartheta_{t-1}{∥}^{2}+{\left(\gamma -\gamma_{t-1}\right)}^{2}}{2\alpha }$$

with respect to θ,ϑ,γ. Any efficient minimization procedure can be used. In our experiments, we used a projected gradient descent, the gradient being given by:

(19) $$\begin{array}{ll} \frac{\partial F_{t}}{\partial \theta } & =\sum_{i=1}^{n}\nabla \ell_{t,i}\left(\theta \right)+\frac{\theta -\vartheta }{\gamma }, \end{array}$$

(20) $$\begin{array}{ll} \frac{\partial F_{t}}{\partial \vartheta } & =\frac{\vartheta -\theta }{\gamma }+\frac{\vartheta -\vartheta_{t-1}}{\alpha }, \end{array}$$

(21) $$\begin{array}{ll} \frac{\partial F_{t}}{\partial \gamma } & =\frac{\Gamma^{2}n}{2}-\frac{{∥\theta -\vartheta ∥}^{2}}{2\gamma^{2}}+\frac{\gamma -\gamma_{t-1}}{\alpha }. \end{array}$$

Note that even though we do not *stricto sensu* obtain the minimizer of $F_{t}$, we can get arbitrarily close to it by taking a large enough number of steps. The main difference between this algorithm and the strategy suggested in [29] is that it is obtained by applying the general proximal update introduced in Equation (7), while they decoupled the update for the initialization step and the learning rate.

### 4.4. Experimental Study

In this section we compare simulated data for the numerical performance of OPMS w.r.t learning the task in isolation with online gradient descent (I-OGA). To measure the impact of learning the gradient step γ, we also introduce mean-OPMS that uses the same strategy as OPMS but only learns the starting point ϑ (it is thus close to [27]). We present the results for regression tasks with the mean-squared-error loss, and then for classification with the hinge loss. The notebooks of the experiments can be found online: https://dimitri-meunier.github.io/ (accessed on 26 September 2021).

#### 4.4.1. Synthetic Regression

**Figure 2:**
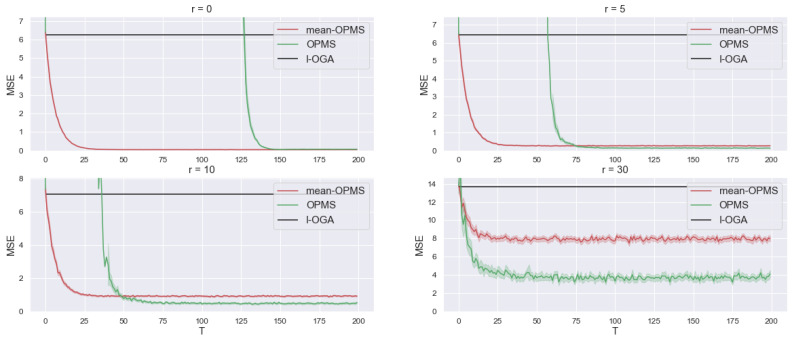
Performance of learning in isolation with OGA (**I-OGA**), OPMS to learn initialization (**mean-OPMS**) and OPMS to learn initialization and step size (**OPMS**). We report the average end-of-task MSE losses at the end of each task, for different values of the task-similarity index r∈{0,5,10,30}. The results are averaged over 50 independent runs to get confidence intervals.

At each round t=1,…,T, the meta learner sequentially receives a regression task that corresponds to a dataset ${\left(x_{t,i},y_{t,i}\right)}_{i=1,\ldots ,n}$ generated as $y_{t,i}=x_{t,i}^{T}\theta_{t}+\epsilon_{t,i}$, $x_{t,i}\in R^{d}$. The noise is $\epsilon_{t,i}∼U\left(\left[-\sigma^{2},\sigma^{2}\right]\right)$ and the $\epsilon_{t,i}$ are all independent, the inputs are uniformly sampled on the (d−1)-unit sphere $S^{d-1}$ and $\theta_{t}=ru+\theta_{0}$, $u∼U\left(S^{d-1}\right)$, $\theta_{0}\in R^{d}$, $r\in R_{+}$. We take d=20, n=30, T=200, $\sigma^{2}=0.5$ and $\theta_{0}$ with all components equal to 5. In this setting, $\theta_{0}$ is a common bias between the tasks, $\sigma^{2}$ is the inter-task variance and *r* characterizes the tasks similarity. We experiment with different values of r∈{0,5,10,30} to observe the impact of task similarity on the meta-learning process. The smaller *r*, the closer are the tasks and for the extreme case of r=0 the tasks are identical, in the sense that the parameters $\theta_{t}$ of the tasks are all the same. We draw attention to the fact that a cross-validation procedure to select α (the parameter of OGMS or OPMS, see Equation (5)) or γ is not valid in the online settings, as it would require having knowledge of several tasks in advance for the former and several datapoints in advance for each task for the latter. Moreover, the theoretical values are based on worst-case analysis and lead in practice to slow learning. In practice, to set these values to the correct order of magnitude without adjusting the constants led to better results. So, for mean-OPMS and OPMS we set $\alpha =1/\sqrt{T}$, for OPMS and I-OGA we set $\gamma =1/\sqrt{n}$. Instead of cross-validation, one can launch several online learners in parallel with different parameter values to pick the best one (or aggregate them). That is the strategy we use to select Γ for OPMS. Note that the exact value of Γ is usually unkown in practice; its automatic calibration is an important open question. To solve (18), after each task we use the exact solution for mean-OPMS and projected Newton descent with 10 steps for OPMS. We observed that not reaching the exact solution of (18) does not harm the performance of the algorithm and 10 steps are sufficient to reach convergence. The results are displayed in Table 1 and Figure 2. On Figure 2, for each task t=1,…,T, we report the average end-of-task loss $MSE_{t}=\sum_{i=1}^{n}\ell_{t,i}\left(\theta_{t,n}\right)/n$ averaged over 50 independent runs (with their confidence intervals). Table 1 reports $MSE_{t}$ averaged over the 100 most recent tasks. The results confirm our theoretical findings: learning γ can bring a substantial benefit over just learning the starting point, which in turn brings a considerable benefit with respect to learning the tasks in isolation. Learning the gradient step makes the meta-learner more robust to task dissimilarities (i.e. when *r* increases) as shown in Figure 2. In the regime where *r* is low, learning the gradient step does not help the meta-learner as it takes more steps to reach convergence. Overall both meta learners are consistently better than learning the task in isolation since the number of observation per task is low.

#### 4.4.2. Synthetic Classification

At each round t=1,…,T, the meta learner sequentially receives a binary classification task with the Hinge loss that corresponds to a dataset ${\left(x_{t,i},y_{t,i}\right)}_{i=1,\ldots ,n}$. The binary labels {−1,1} are generated as a logistic model $P\left(y=1\right)={\left(1+\exp\left(-x^{t}\theta_{t}\right)\right)}^{-1}$. The task parameters $\theta_{t}$ and the inputs are generated as in the regression setting. To add some noise, we shuffle 10% of the labels. We take d=10, n=100, T=500, r=2. For mean-OPMS and OPMS we set $\alpha =1/\sqrt{T}$, for OPMS and I-OGA we set $\gamma =1/\sqrt{n}$. For the optimisation of $F_{t}$ (18) with both OPMS and mean-OPMS we use a projected gradient descent with 50 steps.

**Figure 3:**
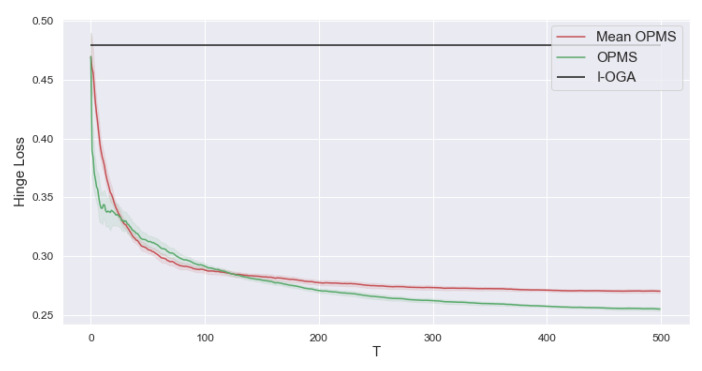
Performance of learning in isolation with OGA (**I-OGA**), OPMS to learn the initialization (**mean-OPMS**) and OPMS to learn the initialization and step size (**OPMS**) on a sequence of classification tasks with the Hinge loss. We report the meta-regret of the Hinge loss. The results are averaged over 10 independent runs (dataset generation) to get confidence intervals.

On Figure 3, for each task t=1,…,T, we report the regret on the end-of-task losses: $R\left(t\right)=\frac{1}{nt}\sum_{k=1}^{t}\sum_{i=1}^{n}\ell_{k,i}\left(\theta_{k,n}\right)$, averaged over 10 independent runs (with their confidence intervals). As the for regression setting, the results confirm our theoretical findings: by learning γ (OPMS), we reach a better overall performance than just learning the initialization (mean-OPMS) and a substantially stronger than independent task learning (I-OGA). Note that, in the classification regime, there is no known closed formed expression for the meta-gradient; therefore, OGMS cannot be used.

## 5. Second Example: Learning the Prior or the Learning Rate in Exponentially Weighted Aggregation

In this section, we will study a generalized Bayesian method, exponentially weighted aggregation. Consider a *finite* set $\Theta_{0}=\left\{\theta_{1},\ldots ,\theta_{M}\right\}\subset R^{d}$. EWA depends on a prior distribution π on $\Theta_{0}$, and on a learning rate η>0, and returns a decision in $\Theta =\mathrm{conv}(\theta_{1},\ldots ,\theta_{M})$ the convex envelope of $\Theta_{0}$. In this section, we work under the following condition.

**Assumption** **3.** There is a $B\in R_{+}^{*}$, such that for any (t,i)∈{1,…,T}×{1,…,n}, the function $\ell_{t,i}$ is Θ→[0,B] and convex.

We will sometimes use a stronger assumption.

**Assumption** **4.** There is a $C\in R_{+}^{*}$, such that for any (t,i)∈{1,…,T}×{1,…,n}, the function $\theta \mapsto \exp(-\ell_{t,i}\left(\theta \right)/C)$ is concave.

Examples of a situation in which Assumption 4 is satisfied are provided in [3]. Note that Assumption 4 implies Assumption 3.

### 5.1. Reminder on EWA

The update in EWA is given by:

(22) $$\theta_{t,i}=\sum_{\theta \in \Theta_{0}}p_{t,i}\left(\theta \right)\theta$$

where $p_{t,i}$ are weights defined by

(23) $$p_{t,i}\left(\theta \right)=\frac{\exp\left[-\eta \sum_{j=1}^{i-1}\ell_{t,j}\left(\theta \right)\right]\pi \left(\theta \right)}{\sum_{\vartheta \in \Theta_{0}}\exp\left[-\eta \sum_{j=1}^{i-1}\ell_{t,j}\left(\vartheta \right)\right]\pi \left(\vartheta \right)}.$$

The strategy is studied in detail in [3]. We refer the reader to [36] and the references therein for connections to Bayesian inference. We recall the following regret bounds from [3]. First, under Assumption 3,

(24) $$\sum_{i=1}^{n}\ell_{t,i}\left(\theta_{t,i}\right)\leq \min_{\theta \in \Theta_{0}}\left[\sum_{i=1}^{n}\ell_{t,i}\left(\theta \right)+\frac{\eta nB^{2}}{8}+\frac{\log\frac{1}{\pi (\theta )}}{\eta }\right].$$

Moreover, under the stronger Assumption 4,

(25) $$\sum_{i=1}^{n}\ell_{t,i}\left(\theta_{t,i}\right)\leq \min_{\theta \in \Theta_{0}}\left[\sum_{i=1}^{n}\ell_{t,i}\left(\theta \right)+C\log\frac{1}{\pi (\theta )}\right].$$

In Section 5.2, we work in the general setting (Assumption 3), and we use our meta-strategy OPMS or OGMS to learn η. In Section 5.3, we use OPMS or OGMS to learn π under Assumption 4.

### 5.2. Learning the Rate η

Consider the uniform prior π(θ)=1/M for any $\theta \in \Theta_{0}$. Then, the regret bound (24) becomes:

(26) $$\sum_{i=1}^{n}\ell_{t,i}\left(\theta_{t,i}\right)\leq \min_{\theta \in \Theta_{0}}\sum_{i=1}^{n}\ell_{t,i}\left(\theta \right)+\frac{\eta nB^{2}}{8}+\frac{\log M}{\eta }$$

and it is then possible to optimize it explicitly with respect to η. The value minimizing the bound is $\eta =\left(2/B\right)\sqrt{2\log(M)/n}$ and the regret bound becomes:

(27) $$\sum_{i=1}^{n}\ell_{t,i}\left(\theta_{t,i}\right)\leq \min_{\theta \in \Theta_{0}}\sum_{i=1}^{n}\ell_{t,i}\left(\theta \right)+B\sqrt{\frac{n\log M}{2}}.$$

In practice, however, while it is often reasonable to assume that the loss function is bounded (as in Assumption 3), very often one does not know a tight upper bound. Thus, one may use a constant *B* that satisfies Assumption 3, but that is far too large. Even though one does not know a better upper bound than *B*, one would like a regret bound that depends on the tightest possible upper bound.

In the meta-learning framework, define:

(28) $$L_{t}\left(\eta \right)=\min_{\theta \in \Theta_{0}}\sum_{i=1}^{n}\ell_{t,i}\left(\theta \right)+\frac{\eta n{\left[\max_{\vartheta \in \Theta_{0},1\leq i\leq n}\ell_{t,i}\left(\vartheta \right)\right]}^{2}}{8}+\frac{\log M}{\eta }$$

for η∈Λ=[1/n,1]. It is immediately necessary to prove that this function is convex and *L*-Lipschitz with $L=n^{2}\log\left(M\right)+nB^{2}/8$. So, Assumption 1 is satisfied, allowing for the use of the OPMS or OGMS strategy without needed a tight upper bound on the losses. Note that, in this context, the OGMS strategy is given by:

$$\eta_{t}=\frac{1}{n}\vee \left[\eta_{t-1}-\alpha \left(\frac{n{\left[\max_{\theta \in \Theta_{0},1\leq i\leq n}\ell_{t,i}\left(\theta \right)\right]}^{2}}{8}-\frac{\log M}{\eta_{t-1}^{2}}\right)\right]\wedge 1.$$

**Theorem** **2.** *Under Assumption 3, using OGMS or OPMS on $L_{t}\left(\eta \right)$, as in (28) with $\eta_{1}=1$, $L=n^{2}\log\left(M\right)+nB^{2}/8$ and*

(29) $$\alpha =\frac{1}{L}\sqrt{\frac{2}{T}}$$

*we have*

(30) $$\begin{array}{ll} \sum_{t=1}^{T}\sum_{i=1}^{n}\ell_{t,i}\left(\theta_{t,i}^{\eta_{t}}\right)\leq \sum_{t=1}^{T}\min_{\theta \in \Theta_{0}}\sum_{i=1}^{n}\ell_{t,i}\left(\theta \right) & +bT\sqrt{\frac{n\log(M)}{2}}\\ & +T\log\left(M\right)+\frac{b^{2}T}{8}+\left(n^{2}\log M+\frac{nB^{2}}{8}\right)\sqrt{2T} \end{array}$$

*where*

(31) $$b=\max_{\theta \in \Theta_{0},1\leq t\leq T,1\leq i\leq n}\left|\ell_{t,i}\left(\theta \right)\right|.$$

Let us compare learning in isolation with meta-learning in this context. When learning in isolation, the hyperparameter η is fixed (as in (2)). If we fix it to the value $\eta_{0}=\left(2/B\right)\sqrt{2\log(M)/n}$ as in (27), the meta-regret is in $BT\sqrt{n\log(M)/2}$. On the other hand, meta-learning leads to a meta-regret in $bT\sqrt{n\log(M)/2}+n^{2}\log M\sqrt{2T}+O\left(nB^{2}\sqrt{T}+T\right)$. In other words, we replace the potentially loose upper bound *B* by the tightest possible bound *b*, at the cost of an additional $n^{2}\log M\sqrt{2T}+O\left(nB^{2}\sqrt{T}+T\right)$ term. Here again, when *T* is large enough with respect to *n*, this term is negligible.

### 5.3. Learning the Prior π

Under Assumption 4, we have the regret bound in (25). Without any information on $\Theta_{0}$, it seems natural to use the uniform prior π on $\Theta_{0}=\left\{\theta_{1},\ldots ,\theta_{M}\right\}$, which leads to

(32) $$\sum_{i=1}^{n}\ell_{t,i}\left(\theta_{t,i}\right)\leq \min_{\theta \in \Theta_{0}}\sum_{i=1}^{n}\ell_{t,i}\left(\theta \right)+C\log M.$$

If some additional information was available, such as, for example: “the best θ is always either $\theta_{1}$ or $\theta_{2}$”, one would rather chose the uniform prior on $\left\{\theta_{1},\theta_{2}\right\}$, and obtain the bound:

(33) $$\sum_{i=1}^{n}\ell_{t,i}\left(\theta_{t,i}\right)\leq \min_{\theta \in \Theta_{0}}\sum_{i=1}^{n}\ell_{t,i}\left(\theta \right)+C\log2.$$

Unfortunately, such information is generally not available. However, in the context of meta-learning, we can take advantage of the previous tasks to learn such information.

Thus, let us define, for any task *t*,

(34) $$\theta_{t}^{*}=\underset{\theta \in \Theta_{0}}{\mathrm{argmin}}\sum_{i=1}^{n}\ell_{t,i}\left(\theta \right)$$

and

(35) $$L_{t}\left(\pi \right)=\sum_{i=1}^{n}\ell_{t,i}\left(\theta_{t}^{*}\right)+C\log\frac{1}{\pi (\theta_{t}^{*})}$$

for $\pi =(\pi \left(\theta_{1}\right),\ldots ,\pi \left(\theta_{M}\right))\in \Lambda$ with

(36) $$\Lambda =\left\{x\in {\left(R_{+}\right)}^{M}:\;\sum_{h=1}^{M}x_{h}=1\;\mathrm{and}\;x_{h}\geq \frac{1}{2M}\right\}.$$

It is important to check that $L_{t}$ is convex and *L*-Lipschitz with L=2CM on Λ; this allows us to use OPMS (or OGMS).

**Theorem** **3.** *Under Assumption 4, using OPMS on $L_{t}\left(\pi \right)$ as in (35) with $\pi_{1}=\left(1/M,\ldots ,1/M\right)$, L=2CM and*

(37) $$\alpha =\frac{1}{2CM\sqrt{T}},$$

define $I^{*}=\left\{\theta_{1}^{*},\ldots ,\theta_{T}^{*}\right\}$ where each $\theta_{t}^{*}$ is as in (34) and $m^{*}=\mathrm{card}\left(I^{*}\right)$. We have

(38) $$\sum_{t=1}^{T}\sum_{i=1}^{n}\ell_{t,i}\left(\theta_{t,i}^{\pi_{t}}\right)\leq \sum_{t=1}^{T}\sum_{i=1}^{n}\ell_{t,i}\left(\theta_{t}^{*}\right)+CT\log\left(2m^{*}\right)+2CM\sqrt{T}.$$

When learning in isolation with a uniform prior, the meta-regret is TClog(M). On the other hand, if $m^{*}$ is small (that is, many of the $\theta_{i}^{*}$s are similar), meta-learning leads to a meta-regret in $CT\log\left(2m^{*}\right)+2CM\sqrt{T}$. For a *T* that is large enough, this is an important improvement.

### 5.4. Discussion on the Continuous Case

Let us now discuss the possibility of meta-learning for generalized Bayesian methods when $\Theta_{0}$ is no longer a finite set. There is a general formula for EWA, given by

(39) $$\rho_{t,i}\left(d\theta \right)=\underset{\rho }{\mathrm{argmin}}\left\{E_{\theta ∼\rho }\left[\sum_{j=1}^{i-1}\ell_{t,j}\left(\theta \right)\right]+\frac{K(\rho ,\pi )}{\eta }\right\}$$

where the minimum is taken over for all probability distributions that are absolutely continuous with π, and where π is a prior distribution, η>0 a learning rate and K is the Kullback–Leibler divergence (KL). Meta-learning for such an update rule is proven in [10,37] but usually does not lead to feasible strategies. Online variational inference [38,39] consists in replacing the minimization on the set of all probability distributions by minimization in a smaller set in order to define a feasible approximation of $\rho_{t,i}$. For example, let ${\left(q_{\mu }\right)}_{\mu \in M}$ be a parametric family of probability distributions, Thus, we define:

(40) $$\mu_{t,i}=\underset{\mu \in M}{\mathrm{argmin}}\left\{E_{\theta ∼q_{\mu }}\left[\sum_{j=1}^{i-1}\ell_{t,j}\left(\theta \right)\right]+\frac{K(q_{\mu },\pi )}{\eta }\right\}.$$

It is discussed in [40] that, generally, when μ is a location-scale parameter and $\ell_{t,j}$ is Γ-Lipschitz and convex, then ${\bar{\ell }}_{t,i}\left(\mu \right):=E_{\theta ∼q_{\mu }}\left[\ell_{t,j}\left(\theta \right)\right]$ is 2Γ-Lipschitz and convex. In this case, under the assumption that $K(q_{\mu },\pi )$ is α-strongly convex in μ, a regret bound for such strategies was derived in [39]:

(41) $$\sum_{i=1}^{n}E_{\theta ∼q_{\mu_{t,i}}}\left[\ell_{t,i}\left(\theta \right)\right]\leq \underset{\mu \in M}{\inf}\left\{E_{\theta ∼q_{\mu }}\left[\sum_{i=1}^{n}\ell_{t,i}\left(\theta \right)\right]+\frac{\eta 4\Gamma^{2}n}{\alpha }+\frac{K(q_{\mu },\pi )}{\eta }\right\}.$$

A complete study of meta-learning of the rate η>0 and of the prior π in this context is an important objective (possibly, with a restriction that $\pi \in \{q_{\mu },\mu \in M\}$). However, this raises many problems. For example, the KL divergence $K(q_{\mu },q_{\mu^{\prime }})$ is not always convex with respect to the parameter $\mu^{\prime }$. In this case, it might help to replace it by a convex relaxation that would allow for the use of OGMS or OPMS. This relates to [41,42], who advocate going beyond the KL divergence in (39); see also [36] and the references therein. This will be the object of future works.

## 6. Proofs

We start with a preliminary lemma that will be used in the proof of Proposition 1.

**Lemma** **1.** *Let a,b,c be three vectors in $R^{p}$. Then:*

(42) $${\left(a-b\right)}^{T}\left(b-c\right)=\frac{{∥a-c∥}^{2}{-∥a-b∥}^{2}-{∥b-c∥}^{2}}{2}.$$

**Proof.**  expand ${∥a-c∥}^{2}={∥a∥}^{2}+{∥c∥}^{2}-2a^{T}c$ in the r.h.s, as well as ${∥a-b∥}^{2}$ and ${∥b-c∥}^{2}$. Then simplify. □

We now prove Proposition 1 separately for the general OGMS strategy, and then for OGMS.

**Proof** **of** **Proposition** **1** **for** **OPMS.** As mentioned earlier, this strategy is an application to the meta-learning setting of implicit online learning [32,33]. We follow here a proof from Chapter 11 in [3]. We refer the reader to [43] and the references therein for tighter bounds under stronger assumptions.

First, $\lambda_{t}$ is defined as the minimizer of a convex function in (5). So, the subdifferential of this function at $\lambda_{t}$ contains 0. In other words, there is a $z_{t}\in \partial L_{t-1}\left(\lambda_{t}\right)$, such that

(43) $$z_{t}=\frac{\lambda_{t-1}-\lambda_{t}}{\alpha }.$$

By convexity, for any λ, for any $z\in \partial L_{t-1}\left(\lambda_{t}\right)$,

(44) $$L_{t-1}\left(\lambda \right)\geq L_{t-1}\left(\lambda_{t}\right)+{\left(\lambda -\lambda_{t}\right)}^{T}z.$$

The choice $z=z_{t}$ gives:

(45) $$L_{t-1}\left(\lambda \right)\geq L_{t-1}\left(\lambda_{t}\right)+\frac{{\left(\lambda -\lambda_{t}\right)}^{T}\left(\lambda_{t-1}-\lambda_{t}\right)}{\alpha },$$

that is,

(46) $$\begin{array}{ll} L_{t-1}\left(\lambda_{t}\right) & \leq L_{t-1}\left(\lambda \right)+\frac{{\left(\lambda -\lambda_{t}\right)}^{T}\left(\lambda_{t}-\lambda_{t-1}\right)}{\alpha }\\ \; & =L_{t-1}\left(\lambda \right)+\frac{∥\lambda -\lambda_{t-1}{∥}^{2}-{∥\lambda -\lambda_{t}∥}^{2}}{2\alpha }-\frac{∥\lambda_{t}-\lambda_{t-1}{∥}^{2}}{2\alpha }\\ \; & =L_{t-1}\left(\lambda \right)+\frac{∥\lambda -\lambda_{t-1}{∥}^{2}-{∥\lambda -\lambda_{t}∥}^{2}}{2\alpha }-\alpha \frac{∥z_{t}{∥}^{2}}{2} \end{array}$$

where we used Lemma 1. Then, note that

(47) $$\begin{array}{ll} L_{t-1}\left(\lambda_{t-1}\right) & =L_{t-1}\left(\lambda_{t}\right)+\left[L_{t-1}\left(\lambda_{t-1}\right)-L_{t-1}\left(\lambda_{t}\right)\right]\\ \; & \leq L_{t-1}\left(\lambda_{t}\right)+∥\lambda_{t-1}-\lambda_{t}∥L\\ \; & \leq L_{t-1}\left(\lambda_{t}\right)+\alpha ∥z_{t}∥L. \end{array}$$

Combining this inequality with (46) gives

(48) $$L_{t-1}\left(\lambda_{t-1}\right)\leq L_{t-1}\left(\lambda \right)+\frac{∥\lambda -\lambda_{t-1}{∥}^{2}-{∥\lambda -\lambda_{t}∥}^{2}}{2\alpha }+\alpha \left(∥z_{t}∥L-\frac{∥z_{t}{∥}^{2}}{2}\right).$$

Now, for any x∈R, $-x^{2}/2+xL-L^{2}/2\leq 0$. In particular, $∥z_{t}∥L-∥z_{t}{∥}^{2}/2\leq L^{2}/2$ and so the above can be rewritten:

(49) $$L_{t-1}\left(\lambda_{t-1}\right)\leq L_{t-1}\left(\lambda \right)+\frac{∥\lambda -\lambda_{t-1}{∥}^{2}-{∥\lambda -\lambda_{t}∥}^{2}}{2\alpha }+\frac{\alpha L^{2}}{2}.$$

Summing the inequality for t=2 to T+1 leads to:

(50) $$\sum_{t=1}^{T}L_{t}\left(\lambda_{t}\right)\leq \sum_{t=1}^{T}L_{t}\left(\lambda \right)+\frac{∥\lambda -\lambda_{1}{∥}^{2}-{∥\lambda -\lambda_{T+1}∥}^{2}}{2\alpha }+\frac{\alpha TL^{2}}{2}.$$

This ends the proof. □

**Proof** **of** **Proposition** **1** **for** **OGMS.** The beginning of the proof follows the proof of Theorem 11.1 in [3].

Note that we can rewrite (4) as

$$\left\{\begin{array}{c} {\tilde{\lambda }}_{t}=\lambda_{t-1}-\alpha \nabla L_{t-1}\left(\lambda_{t-1}\right)\\ \lambda_{t}=\Pi_{\Lambda }\left({\tilde{\lambda }}_{t}\right) \end{array}\right.$$

rearranging the first line, we obtain:

(51) $$\nabla L_{t-1}\left(\lambda_{t-1}\right)=\frac{\lambda_{t-1}-{\tilde{\lambda }}_{t}}{\alpha }.$$

By convexity, for any λ,

(52) $$L_{t-1}\left(\lambda \right)\geq L_{t-1}\left(\lambda_{t-1}\right)+{\left(\lambda -\lambda_{t-1}\right)}^{T}\nabla L_{t-1}\left(\lambda_{t-1}\right)$$

(53) $$\begin{array}{ll} \; & =L_{t-1}\left(\lambda_{t-1}\right)+\frac{{\left(\lambda -\lambda_{t-1}\right)}^{T}\left(\lambda_{t-1}-{\tilde{\lambda }}_{t}\right)}{\alpha }, \end{array}$$

that is,

(54) $$L_{t-1}\left(\lambda_{t-1}\right)\leq L_{t-1}\left(\lambda \right)-\frac{{\left(\lambda -\lambda_{t-1}\right)}^{T}\left(\lambda_{t-1}-{\tilde{\lambda }}_{t}\right)}{\alpha }.$$

Lemma 1 gives:

$$\begin{array}{ll} {\left(\lambda -\lambda_{t-1}\right)}^{T}\left(\lambda_{t-1}-{\tilde{\lambda }}_{t}\right) & =\frac{∥\lambda -{\tilde{\lambda }}_{t}{∥}^{2}-∥\lambda -\lambda_{t-1}{∥}^{2}-{∥\lambda_{t-1}-{\tilde{\lambda }}_{t}∥}^{2}}{2} \end{array}$$

(55) $$\begin{array}{ll} \; & =\frac{∥\lambda -{\tilde{\lambda }}_{t}{∥}^{2}-∥\lambda -\lambda_{t-1}{∥}^{2}-\alpha^{2}{∥\nabla L_{t-1}\left(\lambda_{t-1}\right)∥}^{2}}{2} \end{array}$$

(56) $$\begin{array}{ll} \; & \geq \frac{∥\lambda -\lambda_{t}{∥}^{2}-∥\lambda -\lambda_{t-1}{∥}^{2}-\alpha^{2}{∥\nabla L_{t-1}\left(\lambda_{t-1}\right)∥}^{2}}{2}, \end{array}$$

the last step being justified by:

(57) $$∥\lambda -{\tilde{\lambda }}_{t}{∥}^{2}\geq ∥\lambda -\Pi_{\Lambda }\left({\tilde{\lambda }}_{t}\right){∥}^{2}={∥\lambda -\lambda_{t}∥}^{2}$$

for any λ∈Λ. Plug (55) in (54) to get:

(58) $$L_{t-1}\left(\lambda_{t-1}\right)\leq L_{t-1}\left(\lambda \right)+\frac{∥\lambda -\lambda_{t-1}{∥}^{2}-{∥\lambda -\lambda_{t}∥}^{2}}{2\alpha }+\frac{\alpha ∥\nabla L_{t-1}\left(\lambda_{t-1}\right){∥}^{2}}{2}$$

and the Lipschitz assumption gives:

(59) $$L_{t-1}\left(\lambda_{t-1}\right)\leq L_{t-1}\left(\lambda \right)+\frac{∥\lambda -\lambda_{t-1}{∥}^{2}-{∥\lambda -\lambda_{t}∥}^{2}}{2\alpha }+\frac{\alpha L^{2}}{2}$$

sum the inequality for t=2 to T+1 to get:

(60) $$\sum_{t=1}^{T}L_{t}\left(\lambda_{t}\right)\leq \sum_{t=1}^{T}L_{t}\left(\lambda \right)+\frac{∥\lambda -\lambda_{1}{∥}^{2}-{∥\lambda -\lambda_{T+1}∥}^{2}}{2\alpha }+\frac{\alpha TL^{2}}{2}.$$

This ends the proof of the statement for OGMS. □

We now provide a lemma that will be useful for the proof of Proposition 2.

**Lemma** **2.** *Let G(u,v) be a convex function of (u,v)∈U×V. Define $g\left(u\right)=\inf_{v\in V}G\left(u,v\right)$. Then g is convex.*

**Proof.**  indeed, let λ∈[0,1] and $\left(x,y\right)\in U^{2}$,

(61) $$\begin{array}{ll} g(\lambda x+(1-\lambda )y) & =\underset{v\in V}{\inf}G\left(\lambda x+\left(1-\lambda \right)y,v\right) \end{array}$$

(62) $$\begin{array}{ll} \; & \leq G(\lambda x+\left(1-\lambda \right)y,\lambda x^{\prime }+\left(1-\lambda \right)y^{\prime }) \end{array}$$

(63) $$\begin{array}{ll} \; & \leq \lambda G\left(x,x^{\prime }\right)+\left(1-\lambda \right)G\left(y,y^{\prime }\right) \end{array}$$

where the last two inequalities hold for any $\left(x^{\prime },y^{\prime }\right)\in V^{2}$. Let us now take the infimum with respect to $\left(x^{\prime },y^{\prime }\right)\in V^{2}$ in both sides, this gives:

(64) $$g(\lambda x+(1-\lambda )y)\leq \underset{x^{\prime }\in V}{\inf}\lambda G\left(x,x^{\prime }\right)+\underset{y^{\prime }\in V}{\inf}\left(1-\lambda \right)G\left(y,y^{\prime }\right)$$

(65) $$\begin{array}{ll} \; & =\lambda g(x)+(1-\lambda )g(y), \end{array}$$

that is, *g* is convex. □

**Proof** **of** **Proposition** **2.** Apply Lemma 2 to u=(ϑ,γ), v=θ, U=Λ, V=Θ and

(66) $$G\left(u,v\right)=\sum_{i=1}^{n}\ell_{i,t}\left(\theta \right)+\frac{\gamma \Gamma^{2}n}{2}+\frac{{∥\vartheta -\theta ∥}^{2}}{2\gamma }.$$

This shows $g\left(u\right)=L_{t}\left(\left(\vartheta ,\gamma \right)\right)$ is convex with respect (ϑ,γ). Additionally, *G* is differentiable w.r.t u=(ϑ,γ), so

(67) $$\frac{\partial G}{\partial \vartheta }=\frac{\vartheta -\theta }{\gamma },\;\mathrm{and}\;\frac{\partial G}{\partial \gamma }=\frac{n\Gamma^{2}}{2}-\frac{{∥\vartheta -\theta ∥}^{2}}{2\gamma^{2}}.$$

As a consequence, for $\left(\theta ,\vartheta \right)\in \Theta^{2}$ and $\underline{\gamma }\leq \gamma \leq \bar{\gamma }$,

(68) $${∥\frac{\partial G}{\partial \vartheta }∥}^{2}\leq \frac{4C^{2}}{{\underline{\gamma }}^{2}},\;\mathrm{and}\;{\left|\frac{\partial G}{\partial \gamma }\right|}^{2}\leq \frac{n^{2}\Gamma^{4}}{4}+\frac{4C^{4}}{{\underline{\gamma }}^{4}}.$$

This leads to

(69) $$\begin{array}{ll} ∥\nabla_{u}G\left(u,v\right)∥ & =\sqrt{{∥\frac{\partial G}{\partial \vartheta }∥}^{2}+{\left|\frac{\partial G}{\partial \gamma }\right|}^{2}} \end{array}$$

(70) $$\begin{array}{ll} \; & \leq \sqrt{\frac{n^{2}\Gamma^{4}}{4}+\frac{4C^{2}}{{\underline{\gamma }}^{2}}+\frac{4C^{4}}{{\underline{\gamma }}^{4}}}=:L, \end{array}$$

that is, for each *v*, G(u,v) is *L*-Lipschitz in *u*. So, $g\left(u\right)=\inf_{v\in V}G\left(u,v\right)$ is *L*-Lipschitz in *u*. □

**Proof** **of** **Theorem** 1. Thanks to the Assumption 2, we can apply Proposition 2. That is, Assumption (A1) is satisfied, and we can apply Proposition 1. This gives:

(71) $$\begin{array}{ll} \sum_{t=1}^{T}\sum_{i=1}^{n}\ell_{t,i}(\theta_{t,i}^{\lambda_{t}})\leq & \underset{\theta_{1},\ldots ,\theta_{T}\in \Theta }{\inf}\underset{(\vartheta ,\gamma )\in \Lambda }{\inf}\{\sum_{t=1}^{T}[\sum_{i=1}^{n}\ell_{t,i}\left(\theta_{t}\right)\\ & +\frac{\gamma \Gamma^{2}n}{2}+\frac{∥\theta_{t}{-\vartheta ∥}^{2}}{2\gamma }]+\frac{\alpha TL^{2}}{2}+\frac{∥\vartheta -\vartheta_{1}{∥}^{2}+{\left|\gamma -\gamma_{1}\right|}^{2}}{2\alpha }\}. \end{array}$$

We use direct bounds for the last two terms: $∥\vartheta -\vartheta_{1}{∥}^{2}\leq 4C^{2}$ and $|\gamma -\gamma_{1}{|}^{2}\leq {\left|\bar{\gamma }-\underline{\gamma }\right|}^{2}\leq {\bar{\gamma }}^{2}=C^{4}$. Then note that

(72) $$\begin{array}{ll} \sum_{t=1}^{T}{∥\theta_{t}-\vartheta ∥}^{2} & =T{∥\vartheta -\frac{1}{T}\sum_{s=1}^{T}\theta_{s}∥}^{2}+\sum_{t=1}^{T}{∥\theta_{t}-\frac{1}{T}\sum_{s=1}^{T}\theta_{s}∥}^{2} \end{array}$$

(73) $$\begin{array}{ll} \; & =T{∥\vartheta -\frac{1}{T}\sum_{s=1}^{T}\theta_{s}∥}^{2}+T\sigma^{2}\left(\theta_{1}^{T}\right). \end{array}$$

Upper bounding the infimum on ϑ in (71) by $\vartheta =\frac{1}{T}\sum_{s=1}^{T}\theta_{s}$ leads to

(74) $$\begin{array}{ll} \sum_{t=1}^{T}\sum_{i=1}^{n}\ell_{t,i}\left(\theta_{t,i}^{\lambda_{t}}\right)\leq & \underset{\theta_{1},\ldots ,\theta_{T}\in \Theta }{\inf}\underset{\gamma \in [\underline{\gamma },\bar{\gamma }]}{\inf}\{\sum_{t=1}^{T}\sum_{i=1}^{n}\ell_{t,i}\left(\theta_{t}\right)+\frac{\gamma \Gamma^{2}nT}{2}\\ & +\frac{T\sigma^{2}\left(\theta_{1}^{T}\right)}{2\gamma }+\frac{\alpha TL^{2}}{2}+\frac{C^{2}\left(4+C^{2}\right)}{2\alpha }\}. \end{array}$$

The right-hand side of (74) is minimized with respect to α if $\alpha =\frac{C}{L}\sqrt{\frac{4+C^{2}}{T}}$, which is the value proposed in the theorem, and we obtain:

(75) $$\sum_{t=1}^{T}\sum_{i=1}^{n}\ell_{t,i}\left(\theta_{t,i}^{\lambda_{t}}\right)\leq \underset{\theta_{1},\ldots ,\theta_{T}\in \Theta }{\inf}\underset{\gamma \in [\underline{\gamma },\bar{\gamma }]}{\inf}\left\{\sum_{t=1}^{T}\sum_{i=1}^{n}\ell_{t,i}\left(\theta_{t}\right)+\frac{\gamma \Gamma^{2}nT}{2}+\frac{T\sigma^{2}\left(\theta_{1}^{T}\right)}{2\gamma }+CL\sqrt{(4+C^{2})T}\right\}.$$

The infimum with respect to γ in the r.h.s is reached for

(76) $$\gamma^{*}=\left(\underline{\gamma }\vee \frac{\sigma (\theta_{1}^{T})}{\Gamma \sqrt{n}}\right)\wedge \bar{\gamma }.$$

First, note that

(77) $$\begin{array}{ll} \frac{\gamma^{*}\Gamma^{2}nT}{2} & \leq \left(\underline{\gamma }\vee \frac{\sigma (\theta_{1}^{T})}{\Gamma \sqrt{n}}\right)\frac{\Gamma^{2}nT}{2} \end{array}$$

(78) $$\begin{array}{ll} \; & \leq \left(\underline{\gamma }+\frac{\sigma (\theta_{1}^{T})}{\Gamma \sqrt{n}}\right)\frac{\Gamma^{2}nT}{2} \end{array}$$

(79) $$\begin{array}{ll} \; & =\frac{\Gamma^{2}Tn^{1-\beta }}{2}+\frac{\sigma \left(\theta_{1}^{T}\right)\Gamma T\sqrt{n}}{2}, \end{array}$$

using $\underline{\gamma }=n^{-\beta }$. Then,

(80) $$\begin{array}{ll} \frac{T\sigma^{2}\left(\theta_{1}^{T}\right)}{2\gamma^{*}} & \leq \frac{T\sigma^{2}\left(\theta_{1}^{T}\right)}{2}\left(\frac{1}{\bar{\gamma }}\vee \frac{\Gamma \sqrt{n}}{\sigma (\theta_{1}^{T})}\right) \end{array}$$

(81) $$\begin{array}{ll} \; & \leq \frac{T\sigma^{2}\left(\theta_{1}^{T}\right)}{2}\left(\frac{1}{\bar{\gamma }}+\frac{\Gamma \sqrt{n}}{\sigma (\theta_{1}^{T})}\right) \end{array}$$

(82) $$\begin{array}{ll} \; & =\frac{T\sigma^{2}\left(\theta_{1}^{T}\right)}{2C^{2}}+\frac{\sigma \left(\theta_{1}^{T}\right)\Gamma T\sqrt{n}}{2} \end{array}$$

(83) $$\begin{array}{ll} \; & \leq \frac{T\sigma (\theta_{1}^{T})}{C}+\frac{\sigma \left(\theta_{1}^{T}\right)\Gamma T\sqrt{n}}{2}, \end{array}$$

using $\bar{\gamma }=C^{2}$ and $\sigma (\theta_{1}^{T})\leq 2C$. Plugging (77), (80) and the definition of *L* into (75) gives

(84) $$\begin{array}{ll} \sum_{t=1}^{T}\sum_{i=1}^{n}\ell_{t,i}\left(\theta_{t,i}^{\lambda_{t}}\right) & \leq \underset{\theta_{1},\ldots ,\theta_{T}\in \Theta }{\inf}\{\sum_{t=1}^{T}\sum_{i=1}^{n}\ell_{t,i}\left(\theta_{t}\right)+C\sqrt{\left(\frac{n^{2}\Gamma^{4}}{4}+4C^{2}n^{2\beta }+4C^{4}n^{4\beta }\right)\left(4+C^{2}\right)T} \end{array}$$

(85) $$\begin{array}{ll} \; & +\frac{\Gamma^{2}Tn^{1-\beta }}{2}+\sigma \left(\theta_{1}^{T}\right)T\left(\Gamma \sqrt{n}+\frac{1}{C}\right)\} \end{array}$$

(86) $$\begin{array}{ll} \; & =\underset{\theta_{1},\ldots ,\theta_{T}\in \Theta }{\inf}\{\sum_{t=1}^{T}\sum_{i=1}^{n}\ell_{t,i}\left(\theta_{t}\right)+C\sqrt{\left(4+C^{2}\right)\left(\frac{n^{2}\Gamma^{4}}{4n^{2\vee 4\beta }}+\frac{4C^{2}n^{2\beta }}{n^{2\vee 4\beta }}+\frac{4C^{4}n^{4\beta }}{n^{2\vee 4\beta }}\right)}n^{1\vee 2\beta }\sqrt{T} \end{array}$$

(87) $$\begin{array}{ll} \; & \;\;\;+\left[\frac{\Gamma^{2}}{2}n^{1-\beta }+\left(\Gamma +\frac{1}{nC}\right)\sigma \left(\theta_{1}^{T}\right)\sqrt{n}\right]T\} \end{array}$$

(88) $$\begin{array}{ll} \; & \leq \underset{\theta_{1},\ldots ,\theta_{T}\in \Theta }{\inf}\{\sum_{t=1}^{T}\sum_{i=1}^{n}\ell_{t,i}\left(\theta_{t}\right)+C\sqrt{\left(4+C^{2}\right)\left(\frac{\Gamma^{2}}{4}+4C^{2}+4C^{4}\right)}n^{1\vee 2\beta }\sqrt{T} \end{array}$$

(89) $$\begin{array}{ll} \; & \;\;\;+\left[\frac{\Gamma^{2}}{2}n^{1-\beta }+\left(\Gamma +\frac{1}{C}\right)\sigma \left(\theta_{1}^{T}\right)\sqrt{n}\right]T\} \end{array}$$

(90) $$\begin{array}{ll} \; & \leq \underset{\theta_{1},\ldots ,\theta_{T}\in \Theta }{\inf}\{\sum_{t=1}^{T}\sum_{i=1}^{n}\ell_{t,i}\left(\theta_{t}\right)+C\left(\Gamma ,C\right)\left[n^{1\vee 2\beta }\sqrt{T}+\left(n^{1-\beta }+\sigma \left(\theta_{1}^{T}\right)\sqrt{n}\right)T\right] \end{array}$$

where we took

(91) $$C\left(\Gamma ,C\right)=\max\left(C\sqrt{\left(4+C^{2}\right)\left(\frac{\Gamma^{2}}{4}+4C^{2}+4C^{4}\right)},\frac{\Gamma^{2}}{2},\Gamma +\frac{1}{C}\right).$$

This ends the proof. □

**Proof** **of** **Theorem** **2.** A direct application of Proposition 1 gives

(92) $$\begin{array}{ll} \sum_{t=1}^{T}\sum_{i=1}^{n}\ell_{t,i}\left(\theta_{t,i}^{\eta_{t}}\right)\leq & \underset{\eta \geq \frac{1}{n}}{\inf}\{\sum_{t=1}^{T}\min_{\theta \in \Theta_{0}}[\sum_{i=1}^{n}\ell_{t,i}\left(\theta \right)\\ & +\frac{\eta n{\left[\max_{\vartheta \in \Theta_{0},1\leq i\leq n}\ell_{t,i}\left(\vartheta \right)\right]}^{2}}{8}+\frac{\log M}{\eta }]+\frac{\alpha TL^{2}}{2}+\frac{{\left(\eta -1\right)}^{2}}{2\alpha }\}. \end{array}$$

Thus, we have

(93) $$\sum_{t=1}^{T}\sum_{i=1}^{n}\ell_{t,i}\left(\theta_{t,i}^{\eta_{t}}\right)\leq \underset{\eta \geq \frac{1}{n}}{\inf}\left\{\sum_{t=1}^{T}\min_{\theta \in \Theta_{0}}\left[\sum_{i=1}^{n}\ell_{t,i}\left(\theta \right)+\frac{\eta nb^{2}}{8}+\frac{\log M}{\eta }\right]+\frac{\alpha TL^{2}}{2}+\frac{{\left(\eta -1\right)}^{2}}{2\alpha }\right\}.$$

Now, plugging in the right-hand side

(94) $$\eta =\frac{1}{n}\vee \left(\frac{2}{b}\sqrt{\frac{2\log M}{n}}\right)\wedge 1,$$

we obtain:

(95) $$\sum_{t=1}^{T}\sum_{i=1}^{n}\ell_{t,i}\left(\theta_{t,i}^{\eta_{t}}\right)\leq \sum_{t=1}^{T}\min_{\theta \in \Theta_{0}}\left[\sum_{i=1}^{n}\ell_{t,i}\left(\theta \right)+\frac{b^{2}}{8}+b\sqrt{\frac{n\log(M)}{2}}+\log\left(M\right)\right]+\frac{\alpha TL^{2}}{2}+\frac{1}{2\alpha }.$$

Now, we see that the value $\alpha =\sqrt{2/(TL^{2})}$ leads to:

(96) $$\sum_{t=1}^{T}\sum_{i=1}^{n}\ell_{t,i}\left(\theta_{t,i}^{\eta_{t}}\right)\leq \sum_{t=1}^{T}\min_{\theta \in \Theta_{0}}\left[\sum_{i=1}^{n}\ell_{t,i}\left(\theta \right)+\frac{b^{2}}{8}+b\sqrt{\frac{n\log(M)}{2}}+\log\left(M\right)\right]+L\sqrt{2T}.$$

Rearranging terms, and replacing *L* by its value,

(97) $$\begin{array}{ll} \sum_{t=1}^{T}\sum_{i=1}^{n}\ell_{t,i}\left(\theta_{t,i}^{\eta_{t}}\right)\leq & \sum_{t=1}^{T}\min_{\theta \in \Theta_{0}}\sum_{i=1}^{n}\ell_{t,i}\left(\theta \right)+bT\sqrt{\frac{n\log(M)}{2}}+\frac{b^{2}T}{8}+T\log\left(M\right)\\ & +\left(n^{2}\log M+\frac{nB^{2}}{8}\right)\sqrt{2T}, \end{array}$$

that is the statement of the theorem. □

**Proof** **of** **Theorem** **3.** An application of Proposition 1 leads to

(98) $$\sum_{t=1}^{T}\sum_{i=1}^{n}\ell_{t,i}\left(\theta_{t,i}^{\pi_{t}}\right)\leq \min_{\pi \in \Lambda }\left\{\sum_{t=1}^{T}\left[\sum_{i=1}^{n}\ell_{t,i}\left(\theta_{t}^{*}\right)+C\log\frac{1}{\pi (\theta_{t}^{*})}\right]+\frac{\alpha TL^{2}}{2}+\frac{∥\pi -\pi_{1}{∥}^{2}}{2\alpha }\right\}$$

and so

(99) $$\sum_{t=1}^{T}\sum_{i=1}^{n}\ell_{t,i}\left(\theta_{t,i}^{\pi_{t}}\right)\leq \min_{\pi \in \Lambda }\left\{\sum_{t=1}^{T}\left[\sum_{i=1}^{n}\ell_{t,i}\left(\theta_{t}^{*}\right)+C\log\frac{1}{\pi (\theta_{t}^{*})}\right]+\frac{\alpha TL^{2}}{2}+\frac{1}{2\alpha }\right\}$$

define $\pi_{I^{*}}$ such that $\pi_{I^{*}}\left(\theta_{j}\right)=1/\left(2m^{*}\right)$ if $j\in I^{*}$ and $\pi_{I^{*}}\left(\theta_{j}\right)=1/\left(2\left(M-m^{*}\right)\right)$ otherwise. We have $\pi_{I}^{*}\in \Lambda$ and thus

(100) $$\sum_{t=1}^{T}\sum_{i=1}^{n}\ell_{t,i}\left(\theta_{t,i}^{\pi_{t}}\right)\leq \sum_{t=1}^{T}\left[\sum_{i=1}^{n}\ell_{t,i}\left(\theta_{t}^{*}\right)+C\log\left(2m^{*}\right)\right]+\frac{\alpha TL^{2}}{2}+\frac{1}{2\alpha }.$$

Replace *L* and α by their values to get the theorem. □

## 7. Conclusions

We proposed two simple meta-learning strategies together with their theoretical analysis. Our results clearly show an improvement on learning in isolation if the tasks are similar enough. These theoretical findings are confirmed by our numerical experiments. Important questions remain open. In [27], a purely online method is proposed, in the sense that it does not require storing all of the information of the current task. In the case of OGA, this method allows us to learn the starting point. However, its application to learn the step size is not direct [28]. An important question is, then: is there a purely online method that would provably improve on learning in isolation in this case? Another important question is the automatic calibration of Γ. However, as mentioned in Section 5, we believe that a very general and efficient meta-learning method for learning priors in Bayesian statistics (or in generalized Bayesian inference) would be extremely valuable in practice. 

## Figures and Tables

**Table 1:** Average end-of-task MSE of the 100 last tasks (averaged over 50 independent runs).

	r = 0	r = 5	r = 10	r = 30
I-OGA	6.24	6.44	7.06	13.60
mean OPMS	0.05	0.27	0.93	7.93
OPMS	0.07	0.15	0.49	3.72

## Data Availability

Not applicable.
